# Activity-Based Prospective Memory in Insomniacs

**DOI:** 10.3390/s24113612

**Published:** 2024-06-03

**Authors:** Miranda Occhionero, Lorenzo Tonetti, Federica Giudetti, Vincenzo Natale

**Affiliations:** Department of Psychology “Renzo Canestrari”, University of Bologna, 40127 Bologna, Italy; lorenzo.tonetti2@unibo.it (L.T.); federica.giudetti3@unibo.it (F.G.); vincenzo.natale@unibo.it (V.N.)

**Keywords:** actigraphy, insomnia, prospective memory, sleep

## Abstract

Objective: To investigate the activity-based prospective memory performance in patients with insomnia, divided, on the basis of actigraphic evaluation, into sleep onset, maintenance, mixed and negative misperception insomnia. Methods: A total of 153 patients with insomnia (I, 83 females, mean age + SD = 41.37 + 16.19 years) and 121 healthy controls (HC, 78 females, mean age + SD = 36.99 + 14.91 years) wore an actigraph for one week. Insomnia was classified into sleep onset insomnia (SOI), maintenance insomnia (MaI), mixed insomnia (MixI) and negative misperception insomnia (NMI). To study their activity-based prospective memory performance, all the participants were required to push the actigraph event marker button twice, at bedtime (task 1) and at get-up time (task 2). Results: Only patients with maintenance and mixed insomnia had a significantly lower accuracy in the activity-based prospective memory task at get-up time compared with the healthy controls. Conclusion: The results show that maintenance and mixed insomnia involve an impaired activity-based prospective memory performance, while sleep onset and negative misperception insomnia do not seem to be affected. This pattern of results suggests that the fragmentation of sleep may play a role in activity-based prospective memory efficiency at wake-up in the morning.

## 1. Introduction

In 1990, Einstein and McDaniel [1] gave the first and most cited definition of prospective memory (ProM), that is, memory that concerns the maintenance, retrieval and execution of a previously formed intention. Prospective memory includes two components, remembering that an intention must be carried out (prospective component) and remembering what must be carried out and when (retrospective component), two interconnected but functionally distinct components [2,3,4]. Based on the characteristics of the execution, ProM is classified into three sub-types: time-based ProM (the intention must be completed at a prespecified time [5,6]), event-based ProM (the execution of the intention is linked to an external cue [7]) and activity-based ProM (the intention needs to be retrieved and executed before or after completing a particular activity [8]). Furthermore, the correct execution of a prospective task depends on a series of other factors involving both memory and high-order cognitive functions such as attention [9], executive control [10] and levels of vigilance [11]. Sleep plays a key role in relation to this complex cognitive efficiency.

The relationship between sleep and memory has a long research tradition that began when Jenkins and Dallenbach [12] concluded that sleep has an important, albeit passive, role in memory consolidation by reducing retroactive interference. In more recent times, as Rasch and Born [13] recall, “sleep is a brain state optimizing memory consolidation” (p. 681). A lot of research has demonstrated the active role of sleep in memory processes by reactivating and strengthening associative memory links [14,15,16].

Research that has specifically investigated the ProM–sleep relationship has highlighted how ProM benefits when sleep takes place during the retention interval—between forming and carrying out the intention—and suffers with sleep deprivation (for a review, see [17]). The interpretation of these data puts emphasis on the role of sleep in the mechanism of consolidation of the association between the intention and the context in which it must be carried out [18]. Furthermore, some studies have shown that ProM is influenced by specific sleep features such as sleep quality and sleep stage [19,20]

However, the experimental results do not always agree with each other, particularly when comparing experimental protocols using sleep deprivation with clinical research that studies the effects of sleep disturbance, specifically chronic insomnia, on ProM.

Using the sleep deprivation paradigm, several studies have shed light on the detrimental effect of sleep loss on the ability to perform an intended action at the appropriate times [21,22,23,24]. Sleep deprivation significantly affects ProM through the involvement of specific brain areas, in particular the prefrontal cortex [25], strongly involved in executive and control processes. However, other studies do not document a similar effect of partial sleep deprivation on prospective remembering [17].

The sleep–ProM relationship has also been investigated in the clinical field given the importance of intention memory in daily life (for a review, see [26]). As is known, sleep disturbances represent one of the most widespread problems in the global adult population. Insomnia is defined by difficulties initiating (abnormal sleep onset latency) or maintaining sleep (abnormal waking after sleep onset and numerous nocturnal awakenings) or both conditions [27]. These sleep conditions are accompanied by important daytime impairment and poor subjective quality of sleep [28]. Furthermore, sleep disturbances are associated with neurological dysfunctions, as well as important psychiatric pathologies and cognitive deficits [29].

Several research studies have shown significant impairments in vigilance, memory and psychomotor control [30,31,32] but, on the contrary, other works have found no clear results of cognitive performance impairments in insomniac patients [33,34,35]. With some surprise, a positive relationship between insomnia and cognitive performance has been recently highlighted. For example, Takano et al. [36] demonstrated that people with higher levels of pre-sleep arousal (a typical condition of suffering from insomnia) have a better ability to process sleep-related stimuli with more efficient cognitive performance [37]. In research that examined prospective memory performance using an activity-based task, Tonetti et al. [35] did not detect differences in patients suffering from primary insomnia, while differences emerged only in a population of narcoleptic patients. The authors interpreted the results as related to the specific cognitive problems in this pathology, in terms of memory and the executive processes associated with the well-known alterations of sleep architecture.

In consideration of the critical issue that emerges from the literature, we turn the focus to the ways in which as chronic a condition as insomnia might specifically affect activity-based ProM performance. This research investigated whether sleep quality being compromised in primary insomnia could have consequences for an activity-based prospective memory task. For this purpose, we used the same prospective memory task under two conditions, that is, once before going to bed after a prolonged period of wakefulness and once upon waking up after a period of sleep. To better define the different aspects of primary insomnia, we used actigraphic evaluation. The actigraph has been proven to be sensitive in the diagnosis of insomnia [38]. Furthermore, it has proven to be very important to distinguish between the subjective perception of sleep quality and its objective measurement. As is known, the misperception phenomenon for sleep has been already studied in the clinical literature, highlighting how subjective assessment through sleep diaries may differ from objective measures [39].

The general hypothesis is that there would be an impairment in the execution of both tasks in the sample of insomniacs in comparison to the healthy controls, depending on the consequences that insomnia has for daytime performance in terms of attention and general cognitive functioning.

Moreover, we could put forward the hypothesis that performance in the morning may be different based on the type of insomnia. We could hypothesize that people with insomnia characterized by higher fragmentation of sleep (maintaining and mixed insomnia) perform worse than patients with other types of insomnia. Moreover, sleep onset insomnia, which is characterized by a lengthening of the time to fall asleep, could be associated with an unaffected prospective memory performance due to a sort of “review” of the prospective intention.

## 2. Materials and Methods

### 2.1. Participants

A total of 153 patients with insomnia (I, 83 females, mean age + SD = 41.37 + 16.19 years) and 121 healthy controls (HC, 78 females, mean age + SD = 36.99 + 14.91 years) took part in the current study.

The patients were treated for sleep insomnia at the “Servizio di diagnosi e cura delle insonnie” of the Department of Psychology “Renzo Canestrari”, the University of Bologna. The sample of insomniac patients, based on the actigraphic quantitative criteria put forward by Natale et al. [38,39], was grouped into sleep onset insomnia (SOI) (sleep onset latency > 12 min with the other sleep parameters within the normal limits), maintenance insomnia (MaI) (waking after sleep onset >25 min and/or number of awakenings higher than 5 min > 1.8 but with an SOL within the cut-off values), mixed insomnia (MixI) (both sleep onset latency and waking after sleep onset or a number of awakenings higher than 5 min over the cut-off values) and negative misperception insomnia (NMI) (all actigraphic parameters within the cut-off values). All the patients also complained of difficulty carrying out common daytime activities (such as difficulty concentrating and drowsiness).

The sample of healthy controls (HC, 78 females, mean age + SD = 36.99 + 14.91 years) were recruited at the Laboratory of Applied Chronopsychology of the Department of Psychology “Renzo Canestrari”, the University of Bologna (Bologna, Italy). The control group was selected with the following inclusion criteria: absence of any sleep disorder, no use of drugs that could influence the sleep–wake rhythm. These criteria were verified according to an interview during the recruitment phase.

The samples were balanced by gender (χ21 = 2.9; *p* < 0.09) but not by age (t272 = 2.3; *p* = 0.02).

All the participants were drug-free, aged over 18 years and had no relevant concomitant pathologies or psychiatric diseases, such as schizophrenia or major depressive and/or anxiety disorder, according to the DSM-5 criteria [29].

All the participants (I and HC) gave written informed consent prior to inclusion in the original studies before they underwent home-based actigraphy recording for at least seven consecutive nights, in agreement with the Declaration of Helsinki.

### 2.2. Actigraphy

In the current study, the actigraph model Micro Motionlogger watch (Ambulatory Monitoring, Inc., Ardsley, NY, USA) was used. The hardware consists of a triaxial accelerometer presenting sensitivity equal to or higher than 0.01 g. The sampling frequency is 32 Hz, with the filters set to 2–3 Hz. The software Motionlogger Watchware (Ambulatory Monitoring, Inc., Ardsley, NY, USA) was used to initialize the actigraphs in zero crossing mode to collect motor activity data in 1 min epochs and to download the data onto a PC. Sleep was scored using Action W2.7.1150 software (Ambulatory Monitoring, Inc., Ardsley, NY, USA). Applying validated algorithms [40,41], each epoch was classified as sleep or wake. During the recorded period, participants were asked to fill in a sleep log daily within 30 min from the last morning awakening. Using both the event marker points and the information present in the sleep diaries, scoring was performed by an experienced scorer to determine the time spent in bed.

### 2.3. Actigraphic Sleep/Wake Parameters

The following actigraphic parameters were computed: (1) sleep onset latency (SOL), the time interval in minutes between bedtime and sleep onset (the first minute of the first block of 20 consecutive sleep epochs, including no more than 1 epoch of wake); (2) wake after sleep onset (WASO), the sum in minutes of the wake epochs between sleep onset (SO) and the last sleep offset; (3) number of awakenings longer than 5 min (AWK > 5); (4) sleep motor activity (SMA), mean of motor activity counts in 1 min epochs during the assumed sleep; (5) total sleep time (TST), the sum in minutes of the sleep epochs between SO and the last sleep offset in the morning; (6) sleep efficiency (SE), the ratio between the total sleep time and the time in bed multiplied by 100.

### 2.4. Activity-Based Prospective Memory Task

All the participants were required to remember to push the event marker button of the actigraph before going to bed (task 1) and at get-up time (task 2). With reference to the activity-based PM performance, each actigraphic recording was visually inspected to verify whether the participants remembered to push the event marker button at bedtime and at get-up time, considering only the awakenings in which participants carried out the required task within the first 15 min after the end of sleep [35]. We chose this time range because in an activity-based ProM, the execution times are of primary importance to define the correct performance. In general, ProM models postulate a close association between the cue and target action [42]. For each participant, the percentage of efficiency in tasks 1 and 2 was calculated as follows: number of times one remembers to press the event marker button divided by the number of recording nights multiplied by 100.

## 3. Results

As shown in Table 1, in performing a set of ANCOVAs with age as the covariate, the insomniacs differ from the healthy controls in all the sleep parameters except total sleep time. Furthermore, the prospective performance differs significantly in the two groups for ProM task 2.

To assess the expected differences among the four types of insomnia, we performed an ANCOVA (with age as the covariate) for each actigraphic parameter, the results of which are presented in Table 2.

Regarding the prospective memory performance, we performed two separate ANCOVAs (by inserting age as the covariate) for tasks 1 (bedtime) and 2 (wake-up time), with group as the independent variable. As shown in Table 3, we observed significant differences at get-up time only, i.e., for task 2. The post hoc test (Tukey’s for unequal samples) showed that the performance of the healthy controls was significantly higher in comparison to the performance of those with MaI (*p* = 0.001) and MixI (*p* = 0.002).

## 4. Discussion

The first aim of this study was to determine whether insomnia patients perform worse in an activity-based prospective memory task in comparison to healthy controls. On the basis of our results, we can answer that insomnia patients show lower performance in terms of activity-based prospective memory performance but only in the morning, during the transition between sleep and wake. This result validates the role of sleep in modulating cognitive performance. This result could generate some perplexity since insomnia is considered a disorder with important consequences on global daytime cognitive functioning. In the execution of the prospective task, it is known that the type and importance of the task have great value. According to Walter and Meier [43], the more important the intention is judged to be, the greater the probability that it will be recovered: the effectiveness of the task is strongly conditioned by the relevance of the intention. Furthermore, ongoing activity, i.e., the cognitive load elaborated between the processing and the execution of the intention, is significant considering the sharing of attentional resources to be allocated to the intention and ongoing activity [44]. In activity-based tasks, the prospective pop-up requires few strategic attentional resources, as it is elicited by the contextual conditions with little impairment by ongoing activity. A third but no less important element is linked to the levels of daytime hyperarousal often present in insomniac patients [45,46]. These elements, taken together, could be a valid interpretative key to our data: cognitive tasks of high importance and high ecological adaptation determine a sort of compensation that to some extent reduces the daytime effects of insomnia. In relation to this, the study by Ballesio, Cerolini et al. [47] reported that good sleepers tended to perform slightly better in task switching after a night of disturbed sleep compared to their habitual sleep. The authors argued that engagement in this challenging task, with increased cognitive efforts to compensate for the deficits, may have resulted in more efficient cognitive performance.

As regards the second aim, using actigraphic recording (i.e., an objective measure of the activity–rest cycle), we considered four types of insomnia, i.e., sleep onset insomnia, maintenance insomnia, mixed insomnia and negative misperception insomnia. The results show the worst performance for maintenance and mixed insomnia, with intermediate values for sleep onset and misperception insomnia. This result may underline the role of sleep continuity in improving prospective memory performance. Sleep fragmentation is suggestive of an alteration of physiological alternation in the NREM-REM sleep cycle, indirectly confirming the sequential hypotheses [48,49].

As regards sleep latency as a function of sleep onset insomnia, this study did not show any significant effects on prospective memory. From our data, it would seem that the relationship between sleep and prospective memory could be intrinsically connected to the physiological cyclical sleep pattern during the night. In this regard, it is necessary to reiterate the importance of an instrumental diagnosis which, alongside a subjective report, supports an objective evaluation [38,39].

A separate evaluation should be reserved for sleep onset and negative misperception insomnia. In these conditions, we observed good performance in both tasks. According to Kvavilashvili and Fisher [50], during wakefulness, people can recover their prospective intentions more often, strengthening their representation and therefore their consolidation. Another interpretation of our results could be to consider pre-sleep intrusive mentation (mental rumination) [51]. Sleep onset insomnia is explained as extreme difficulty in detaching oneself from daytime thoughts (cognitive and/or emotional). This mentation conflicts with the necessary relaxation that precedes and determines sleep onset. We think that this pre-sleep cognition could facilitate thoughts concerning the prospective agenda. So, ruminating thinking could have a consolidation effect on the prospective intention, with an improvement in the prospective task on getting up in the morning.

We acknowledge one important limitation of the present study. Since we examined just one type of PM task, it would be appropriate to think of similar studies on other types of prospective memory in relation to different types of engagement of cognitive function in attention and strategic processes.

Despite these limitations, we think that our study remains very relevant based on the use of very suitable instrumentation for the study of sleep both in normal conditions and clinical settings.

## 5. Conclusions

The present study contributes to the still open research question about the detrimental role of different types of insomnia in the modulation of ProM. The main finding is that the specific objective sleep measures allowed us to assess sleep more precisely during the retention interval and to analyze the relationships between different types of insomnia and activity-based ProM. Patients with maintenance and mixed insomnia showed a more impaired ProM performance than patients with sleep onset and misperception insomnia. This pattern of results allows us to conclude that different clinical aspects of insomnia may impact memory processes in a non-homogeneous way. A differentiated approach is necessary both with regard to clinical intervention and in the study of the relationships between sleep and cognitive functions.

The second, important concern is linked to the role of actigraphy within the two-dimensional approach of this study, clinical on the one hand and research-based on the other [52]. From a clinical point of view, actigraphy proves to be an excellent tool for an effective clinical diagnosis of the main and most frequent sleep disorders: diagnosis is significantly improved compared to when using only the history reported by the patient. From the point of view of experimental sleep research, actigraphy allows for selection of the participants with an objective measurement of sleep in ecological conditions and for extended periods. In addition to an ecological and objective sleep assessment, actigraphy can be also profitably used to investigate cognitive efficiency, as shown in the present study, as well as in previous studies [19,35].

## Figures and Tables

**Table 1:** Means and standard deviations of actigraphic sleep measures and ProM tasks in healthy controls (HC) and insomniac (I) patients. Statistics are also reported, with significant effects in italics.

	HC	I	F_(1,271)_	*p*
TST	415.68 ± 43.40	416.27 ± 70.96	0.18	n.s.
SE	94.63 ± 1.63	88.13 ± 10.55	41.41	<0.001
SOL	7.97 ± 2.59	14.79 ± 13.26	31.72	<0.001
WASO	12.60 ± 5.68	41.37 ± 38.51	62.01	<0.001
AWK > 5	1.68 ± 0.91	2.99 ± 1.66	55.90	<0.001
SMA	10.7 ± 2.30	17.14 ± 12.07	30.93	<0.001
Task 1	85.26 ± 22.52	84.24 ± 23.82	0.04	n.s.
Task 2	72.67 ± 29.05	53.72 ± 35.90	18.57	<0.00001

Note: TST = total sleep time (min.); SE = sleep efficiency (%); SOL = sleep onset latency (min.); WASO = wake after sleep onset (min.); AWK > 5 = awakenings lasting more than 5 min (number); SMA = sleep motor activity (counts). Task 1 (%) = accuracy in the prospective memory task at bedtime; Task 2 (%) = accuracy in the prospective memory task at get-up time.

**Table 2:** Means and standard deviations of actigraphic sleep measures in different types of insomniac patients. Statistics are also reported, with significant effects in italics.

	SOI	MaI	MixI	NMI	F_(3,149)_	*p*
TST	438.90 ± 62.90	404.33 ± 59.09	392.44 ± 104.71	438.25 ± 44.74	4.12	<0.01
SE	91.29 ± 2.61	86.62 ± 6.69	80.08 ± 17.55	94.61 ± 2.25	16.86	<0.001
SOL	25.48 ± 8.75	7.81 ± 2.95	28.48 ± 18.38	7.74 ± 2.74	50.83	<0.001
WASO	17.38 ± 8.75	56.56 ± 35.03	65.97 ± 50.57	15.33 ± 5.47	24.46	<0.001
AWK > 5	2.16 ± 0.44	3.78 ± 1.75	3.88 ± 1.60	1.74 ± 0.78	25.36	<0.001
SMA	14.26 ± 4.35	18.72 ± 7.08	25.82 ± 20.70	9.95 ± 2.65	14.59	<0.001

Note: SOI = sleep onset insomnia; MaI = maintenance insomnia; MixI = mixed insomnia; NMI = negative misperception insomnia; TST = total sleep time (min.); SE = sleep efficiency (%); SOL = sleep onset latency (min.); WASO = wake after sleep onset (min.); AWK > 5 = awakenings lasting more than 5 min (number); SMA = sleep motor activity (counts).

**Table 3:** Means and standard deviations of accuracy in the activity-based prospective memory tasks in different types of insomniac patients and the control group. Statistics are also reported, with significant effects in italics.

	SOI	MaI	MixI	NMI	HC	F_(4,268)_	*p*
Task 1	87.01 ± 21.84	81.45 ± 28.69	84.69 ± 16.65	86.02 ± 22.97	85.25 ± 22.51	0.29	n.s.
Task 2	63.99 ± 34.76	49.01 ± 36.51	43.26 ± 34.45	62.51 ± 34.54	72.67 ± 29.05	6.59	<0.001

Note: SOI = sleep onset insomnia; MaI = maintenance insomnia; MixI = mixed insomnia; NMI = negative misperception insomnia; HC = healthy controls; Task 1 (%) = accuracy in the prospective memory task at bedtime; Task 2 (%) = accuracy in the prospective memory task at get-up time.

## Data Availability

The data are not publicly available and cannot be shared due to ethical issues.
